# Sulfonal in Insomnia

**Published:** 1888-11

**Authors:** 


### *SULFONAL IN INSOMNIA.*

A. Cramer (*Neurolog. Centralblatt*, 1888, 430,) reports the results of 407 administrations of sulfonal to forty-five patients with various mental disorders. Thirty times there was no result; 377 times sleep lasting five or more hours was produced, usually one-quarter to one hour after the medicine had been taken. The dose varied from one to three grammes. Unpleasant secondary effects were only observed in one instance, and consisted merely in some sleepliness on the following morning. The author then instituted experiments to determine whether the drug possessed any disturbing influence on the diastasic action of the saliva, and on the power of artificially prepared gastric and pancreatic secretions to digest fibrin. The results showed such power to be absent.

Rabbas (*Berliner klin. Wochenschrift*, 1888, 330,) has also obtained only good results with sulfonal in the insomnia of mental disorders. In doses of two to three grammes it acts better than either amyl hydrate or paraldehyde; and though sleep is produced by chloral more promptly, it does not last so long. He has found the remedy efficient in the worst maniacal conditions where chloral and paraldehyde had proved unavailing. Most of the twenty-seven cases to whom the medicament was given 220 times, were instances of mania and melancholia.—*American Journal Medical Sciences.*

